# Medical Opinion and Movement

**Published:** 1909-11-06

**Authors:** 


### MEDICAL OPINION AND MOVEMENT.

AN interesting account of some pathological research on a fatal case of diphtheria was given by Dr. Guillain at a meeting of the Société Médical des Hopitaux. The case was that of a man aged 23, who, after an attack of pharyngeal diphtheria, developed complete paralysis of the palate and then bulbar paralysis (hiccough, vomiting, bradycardia, and dyspnœa), to which he rapidly succumbed. The cerebro-spinal fluid showed no cellular reaction, and, inoculated into the rabbit, did not produce any trouble. Portions of the bulb were then taken in the region of the nuclei of the nerves, and after being washed with running water for 24 hours and treated with physiological serum they were injected into rabbits. These animals died rapidly, whereas control animals inoculated with other portions of the nervous system were unaffected. At the autopsy the blood from the hearts of these animals was sterile. These experiments would appear to show that the toxin was confined to the bulbar region. The author states that further observations have shown that nervous tissue placed in contact with the diphtheria toxin fixes it, and that even after lavage for 24 hours inoculation of the tissue in animals will determine paralysis and death. The lipoids extracted from the nervous system with ether appear to be specially instrumental in fixing the toxins; but, apart from this interesting question of the fixation of the toxins, this case would seem to demonstrate the cerebral origin of diphtheritic paralyses in man.

AT the annual meeting of the Italian Society of Medicine at Milan, Dr. C. Colombo read a paper advocating the use of the Galvanic Current in Cardiac Cases where it is especially required to raise the muscular tone of the heart; in fact, in any case where cardiac tonics are indicated. The muscular fibres of the heart are affected by the electric current either directly or indirectly through the sympathetic nerves. The effects can easily be confirmed by means of cardiograms taken before and after galvanisation. These effects only last a few hours after the first application, but after 20 or 30 applications the effect is more lasting, or at least the patient is relieved for a large portion of the day. The author admits, however, that after a certain time the effect of the galvanisation diminishes, and it is then desirable to suspend the treatment for two or three weeks and have recourse to cardiac tonics. If galvanic treatment is then resumed again it is found to be as efficacious as at first. The technique of the application is as follows: A large negative pad electrode is placed on the back between the second and eighth dorsal vertebræ. The positive electrode with a surface of 50 to 60 square centimetres is placed on the præcordial region corresponding to the base of the heart. A current is then allowed to pass through, gradually increasing to eight or ten milliamperes. It is then rapidly increased to 40 to 60 milliamperes and immediately reduced again to the original strength. The rapid increase of the current should not last more than one or two seconds, and should be repeated a dozen times every half minute. The application should be made every day at the time when the patient is most distressed.

AT the same meeting Dr. R. Robinson, of Paris, drew attention to a vegetable substance known to the Arabians by the name of "Gamyr," which he finds has the same curative properties in cases of renal disease as tincture of cantharides, but is devoid of the toxic action of the latter drug. Reference has already been made in these columns to the observations of Lancereaux, by which he claimed to demonstrate quite astonishing curative effects of very small doses of tincture of cantharides in cases of Bright's disease. Dr. Robinson believes that equally good results may be obtained with this new drug called "gamyr." Injected into rabbits, it produces oliguria, considerable oxaluria and hyperacidity of the urine. Histological examination of the kidneys shows marked changes in the tubules and Malpighian glomeruli, indicative of an acute epithelial nephritis without involvement of interstitial tissue. In man, under normal conditions, 10 to 12 grammes of this substance induces marked oliguria with oxaluria and casts. On the other hand, if it is administered to a patient suffering from chronic nephritis it produces a considerable polyuria and the albuminuria disappears in a few days. It is evident, therefore, from these observations that this vegetable product "gamyr" is a renal irritant, which, administered with care and in small doses, may prove of considerable utility in certain renal affections.

AT the recent International Congress of Medicine at Budapest Drs. Arnozan and Carles, of Bordeaux, raised an interesting question as to the action of the leucocytes in relation to the absorption and elimination of medicinal substances. It is generally supposed that such substances, when absorbed, circulate in the blood in a state of solution. But recent observations have shown that when such substances as calomel, iodoform, arsenic bisulphate, metallic mercury, and certain iron and silver salts are injected hypodermically, they are taken up by the leucocytes and circulate in the blood in this way. Enclosed in these corpuscles the authors attempt to show the changes of fortune these substances may undergo. Some are modified in composition or decomposed altogether, others are ejected by the corpuscles within certain viscera and tend to accumulate there. Others, again, are eliminated with the corpuscles either at the surface of the intestinal mucous membrane or at some ulcerating surface, if such exists. The authors suggest that these facts explain the varying results obtained with different remedies. In the case, for instance, in which there is an attraction of leucocytes to the seat of disease, one can understand that a very small dose concentrated upon the lesion by the accumulation of leucocytes might have a very pronounced effect. On the other hand, medicaments locked up, as it were, in the bodies of the leucocytes might never encounter the morbid agents, which may also be shut up in other leucocytes or free in the tissues. In this way may be explained the frequent failure of the internal administration of antiseptics in infective cases. It is suggested, further, that in cases of microbic infection too much food or drugs may encumber the leucocytes and disable them from carrying out their functions of absorbing and eliminating the microbes and their toxins from the system.

---

As a result of an extremely able and critical survey of published cases, Dr. A. E. Martin concludes in an article contributed to *Brain* that long Remissions and even Recoveries occur in Tuberculous Meningitis. Further, he concludes that such recoveries are possibly more frequent than is believed, as no fewer than twenty cases have been recorded since 1894 which will bear the most rigorous investigation; and in addition there are many other cases in which the evidence, though not of the nature of proof positive, leaves little doubt that the diagnosis has been correct. Such recoveries are brought about either when the resistance of the patient is so much greater than usual that the disease is checked early in its course, or when the virulence of the bacilli is so much less than usual that the lesion in the meninges becomes localised and later undergoes a fibrous change. Such an arrested lesion may, however, at a later period form the focus of a fresh infection, which usually terminates fatally; consequently prognosis must, even when recovery has apparently occurred, be guarded. Lastly, no treatment has so far produced any specific effect in promoting the favourable termination of the disease. It is disquieting to notice that after analysing the Reports of seven London hospitals from 1897 onwards, Dr. Martin was compelled to reject 21 out of 22 recorded recoveries from this disease—sometimes because the patients had not recovered, sometimes because the diagnosis had never been satisfactorily established. This is distinctly a serious matter, because Hospital Reports are generally regarded as so completely trustworthy as to be beyond suspicion.

---

The relation of Syphilis to Tabes Dorsalis presents a problem which is certainly as yet unsolved. In the *Quarterly Journal of Medicine* a research on this subject by Drs. Judson Bury and A. Ramsbottom is summarised. These authors were confronted by a case of tabes occurring in a woman twenty-two years of age, who, they felt convinced, had never uffered from syphilis. On examining the cerebro-spinal fluid they found no lymphocytosis, thus confirming their conclusion as to syphilis, but raising the old question of whether there can be tabes dorsalis without antecedent syphilis. They then systematically investigated the cerebro-spinal fluid of a series of consecutive cases of tabes, and also of some cases of cerebro-spinal syphilis and of other non-luetic cerebral lesions. The counts were done on a uniform plan, and the results are of considerable interest. Of ten patients suffering from cerebral tumour, Menière's disease, bulbar paralysis, and the like, but not, as far as was known, the subjects of syphilis, no lymphocytosis was found in any. On the other hand six patients with cerebro-spinal syphilis had each a very considerable lymphocytosis. In thirty-four cases of tabes and general paralysis, twenty-nine presented lymphocytosis; of these twenty-seven admitted syphilis, two denied it. Of the remaining five, in which no lymphocytosis occurred, no history of syphilis could be obtained or reasonably assumed. The authors sum up, contrary to the views of Dr. Purves Stewart and many others, that lymphocytosis is not a constant event in tabes and general paralysis; and that, though syphilis may play an important part in the production of these diseases, it is not an essential factor.

---

The Oral Administration of Tuberculin and its possibilities have attracted a good deal of attention lately in the profession, chiefly owing to the papers recommending this treatment read before the Royal Society of Medicine by Drs. Latham and Inman. The conclusions of these two observers were summarised as being chiefly two: that tuberculin (T.R.) administered orally exerts a definite influence on tubercular disease, and that such action bears a constant and inverse relation to the patient's temperature. As these are at variance with the previous researches of other experimenters, Drs. Lawson and Gettings have endeavoured to settle the matter by a new research from which every source of fallacy has been as carefully as possible excluded. They set themselves two questions, and succeeded in answering them. The first was, Can tuberculin given by the mouth exert an action on the blood content, as indicated by the variation of the opsonic index, of a person suffering from tuberculous disease? The answer is that in 64 per cent. of cases a negative phase immediately appears, and therefore they support Latham and Inman. The second was, Is the inverse relation of the temperature curve to that of the opsonic index so constant that the latter, and therefore the varying resistance of the patient to tuberculous disease, may be safely inferred from the former? The answer is that in 55 per cent. such an inverse relation definitely occurs, but that this proportion does not constitute sufficient basis for an affirmative answer to the question. It will be noticed that they take for granted that the opsonic index faithfully reflects the resistance of the patient to microbic invasion, an assumption which, in the case of tuberculosis especially, is still of very doubtful validity.

---

An interesting discussion on the Ætiology of Psoriasis took place at the annual meeting of the American Dermatological Association, and is reported in the *Medical Record*. Dr. Pollitzer, who opened the debate, summed up thus. Rheumatism, gout, neuroses, and heredity are not direct ætiological factors in the production of psoriasis; but it can, in the present state of our knowledge, be neither affirmed nor denied that they may have some bearing on this obscure condition. Psoriasis is one member of a group of parakeratoses, to which seborrhœa corporis and eczema seborrhœicum also belong. It is probably due to an external microbic agent. Dr.

Schamberg, while declining to commit himself to the parasitic theory, formulated three propositions for consideration: that psoriasis may be due to the circulation and deposition of a micro-parasite, analogous to what is observed in syphilis and variola; that it may be the result of the implantation of an exogenous parasite, as are favus, ringworm, and tinea versicolor; that it may be caused by one of the common facultative parasitic organisms when constitutional predisposition favours their development. No one of these is to be lightly brushed aside; there are points which tell in favour of each, as well as others which indicate the reverse. Two or three speakers regarded the parasitic theories as unlikely, for various reasons; but the majority seemed to think that, though unproved, it is in one or other of Schamberg's three forms most probably the true explanation of the ætiology. It was agreed on all hands that much mystery still surrounds the condition, and that much research will be required to dissipate it.

---

The meaning of the somewhat lately introduced term Anaphylaxis has been recently explained in these columns (The Hospital, October 30, p. 125). In the *Journal of Tropical Medicine and Hygiene*, Dr. J. B. Cleland propounds some suggestions as to the *rôle* anaphylaxis may play in the ætiology of certain obscure diseases. Puerperal eclampsia has lately been attributed in America to anaphylaxis following the absorption by the mother of fœtal syncytium at intervals sufficient to allow of toxic results. Dr. Cleland had arrived at some such conclusion independently, but was forestalled in the matter of publication. He now suggests the possibility that anaphylaxis and blackwater fever are causally connected. He supposes that a number of the small free forms of the malarial parasite die naturally, or are killed by quinine; that their protoplasm, after solution in the plasma, sets going the process that may eventuate in the formation of a specific precipitin: that, after an interval sufficiently long to set up anaphylaxis, a second batch likewise die and enter into solution: and that blackwater fever is the resultant condition, the evidence of anaphylaxis to (dead) plasmodium material. This conjecture will, he thinks, explain the *rôle* that quinine has been believed by some to play in determining the onset of hæmoglobinuria: the occurrence of cases in malarial subjects after returning to temperate climates: the rarity of the disease in new arrivals in the tropics: and even its somewhat local incidence.

---

Psoitis, or inflammation of the psoas muscle, a condition which has been discussed recently by Verdun in the *Gazette des Höpitaux*, is rarely of primitive origin, and is usually due to extension from some neighbouring focus of disease. Nearly always unilateral, and in 70 per cent. of cases occurring on the right side, it is most commonly the result of some appendicular trouble. When found on the left side, it is the outcome either of puerperal infection or of disease of the uterine appendages. Puerperal infection and osteomyelitis may bring about psoïtis on either side, though it is rare for both sides to be affected at one time. The muscular lesions are diffuse or localised according as the disease is the result of a generalised or of a localised infection. The muscle is affected in its upper, middle, or lower portions according as the primitive focus of inflammation is nearest respectively to one or other of these. Thus the upper portion will be affected as the result of a perinephritic abscess, the middle when the hypogastric glands are inflamed, and the lower when the bursa between the muscle and the hip-joint is in a state of suppuration. The fact that it is the middle portion which is the most frequently affected is not due to any specific weakness of the musculature, but to the close proximity of the appendix and the internal iliac glands to the psoas muscle at this point. The clinical diagnosis of the condition is a matter of much practical importance, especially in so-called primary or idiopathic psoïtis.

---

In the *Wien. Med. Woch.* Kieusfuchs discusses the diagnosis of intrathoracic goître. Previous to the discovery of the *x*-rays this condition was rarely recognised except during surgical operations or in the post-mortem room. Twelve cases have, however, been reported by Kienböch in which radiography was instrumental in leading to its discovery, in two of which the diagnosis was subsequently confirmed by operation. Three varieties of the condition are met with. In the first the tumour is mainly cervical, but there occurs a prolongation downwards into the superior aperture of the thorax. The second type is half cervical, half intrathoracic. The goître in the third type is mainly or entirely in the thorax. The first two varieties are not rare, and usually can be diagnosed without much difficulty. In view of the fact that exophthalmic symptoms may occur without obvious enlargement of the thyroid gland, it is not easy to diagnose the third variety, and, moreover, the condition being rare, the possibility of its existence is apt to be overlooked. Tumours of this kind vary greatly in size, and may occur in various situations. They may be median, retro-clavicular, retro-sternal, or retro-vasal—that is, they may lie behind the common carotid or innominate arteries. Symptoms and signs, in addition to those due to Graves' disease, depend on the size of the tumour, and may be either absent or exceedingly obscure. The trachea is usually thrust out of position, and may be compressed and ulcerated or twisted. Stridor is sometimes a striking symptom. The presence of dilated veins over the upper half of the thorax and dulness over the sternum may facilitate diagnosis. Dysphagia is much rarer than tracheal obstruction owing to the pliability of the œsophagus. The pressure exerted by the tumour on surrounding structures may give rise to inequality in the pulses, cyanosis, and venous dilatation, especially in the neighbourhood of the sternum, and the sympathetic, vagus, and recurrent laryngeal nerves may be thereby compressed. In three of Kienböch's cases hoarseness was present, and in one under the author's care there was paralysis of the recurrent laryngeal nerve.

